# Pharmacological inhibition of mTORC1 increases CCKBR-specific tumor uptake of radiolabeled minigastrin analogue [^177^Lu]Lu-PP-F11N

**DOI:** 10.7150/thno.45440

**Published:** 2020-08-29

**Authors:** Michal Grzmil, Yun Qin, Carina Schleuniger, Stephan Frank, Stefan Imobersteg, Alain Blanc, Martin Spillmann, Philipp Berger, Roger Schibli, Martin Behe

**Affiliations:** 1 Center for Radiopharmaceutical Sciences, Paul Scherrer Institute, Villigen, Switzerland.; 2 Division of Neuropathology, Institute of Pathology, University of Basel, Switzerland.; 3 Laboratory of Nanoscale Biology, Paul Scherrer Institute, Villigen, Switzerland.; 4 Department of Chemistry and Applied Biosciences, ETH Zurich, Switzerland.

**Keywords:** Cholecystokinin B receptor, minigastrin, PPF-11N, RAD001, peptide receptor radionuclide therapy

## Abstract

**Rationale:** A high tumor-to-healthy-tissue uptake ratio of radiolabeled ligands is an essential prerequisite for safe and effective peptide receptor radionuclide therapy (PRRT). In the present study, we searched for novel opportunities to increase tumor-specific uptake of the radiolabeled minigastrin analogue [^177^Lu]Lu-DOTA-(DGlu)_6_-Ala-Tyr-Gly-Trp-Nle-Asp-Phe-NH_2_ ([^177^Lu]Lu-PP-F11N), that targets the cholecystokinin B receptor (CCKBR) in human cancers.

**Methods:** A kinase inhibitor library screen followed by proliferation and internalization assays were employed to identify compounds which can increase uptake of [^177^Lu]Lu-PP-F11N in CCKBR-transfected human epidermoid carcinoma A431 cells and natural CCKBR-expressing rat pancreatic acinar AR42J cells. Western blot (WB) analysis verified the inhibition of the signaling pathways and the CCKBR level, whereas the cell-based assay analyzed arrestin recruitment. Biodistribution and SPECT imaging of the A431/CCKBR xenograft mouse model as well as histological analysis of the dissected tumors were used for *in vivo* validation.

**Results:** Our screen identified the inhibitors of mammalian target of rapamycin complex 1 (mTORC1), which increased cell uptake of [^177^Lu]Lu-PP-F11N. Pharmacological mTORC1 inhibition by RAD001 and metformin increased internalization of [^177^Lu]Lu-PP-F11N in A431/CCKBR and in AR42J cells. Analysis of protein lysates from RAD001-treated cells revealed increased levels of CCKBR (2.2-fold) and inhibition of S6 phosphorylation. PP-F11N induced recruitment of β-arrestin1/2 and ERK1/2 phosphorylation. In A431/CCKBR-tumor bearing nude mice, 3 or 5 days of RAD001 pretreatment significantly enhanced tumor-specific uptake of [^177^Lu]Lu-PP-F11N (ratio [RAD001/Control] of 1.56 or 1.79, respectively), whereas metformin treatment did not show a significant difference. Quantification of SPECT/CT images confirmed higher uptake of [^177^Lu]Lu-PP-F11N in RAD001-treated tumors with ratios [RAD001/Control] of average and maximum concentration reaching 3.11 and 3.17, respectively. HE staining and IHC of RAD001-treated tumors showed a significant increase in necrosis (1.4% control vs.10.6% of necrotic area) and the reduction of proliferative (80% control vs. 61% of Ki67 positive cells) and mitotically active cells (1.08% control vs. 0.75% of mitotic figures). No significant difference in the tumor vascularization was observed after five-day RAD001 or metformin treatment.

**Conclusions:** Our data demonstrates, that increased CCKBR protein level by RAD001 pretreatment has the potential to improve tumor uptake of [^177^Lu]Lu-PP-F11N and provides proof-of-concept for the development of molecular strategies aimed at enhancing the level of the targeted receptor, to increase the efficacy of PRRT and nuclear imaging.

## Introduction

Overexpression of G-protein coupled receptors (GPCRs) that selectively bind their peptide ligands allowed the development of the peptide receptor radionuclide therapy (PRRT) for human cancers 1. One of the most important properties of PRRT is a high uptake ratio of tumor-to-healthy-tissue in terms of radiolabeled ligands. Consequently, the molecular strategies which increase uptake of radiopharmaceuticals selectively in cancer tissue while sparing healthy organs from cytotoxic side effects have huge potential to improve PRRT efficacy. Activated GPCRs by agonistic ligand-based radiotherapeutics undergo desensitization, whereby arrestin-bound GPCRs can be internalized and trafficked to lysosomes for degradation, or to endosomes for their recycling back to the cell surface 2. As a consequence, ligand-conjugated radioactive nuclides can be delivered into the cancer cells. The cholecystokinin B receptor (CCKBR), which belongs to the GPCR family, is predominantly expressed in the central nervous system and gastrointestinal tract, whereby it is involved in the regulation of neurotransmission in the brain as well as in the production of gastric acid and the differentiation of gastric mucosa cells, respectively 3. Importantly, high expression of CCKBR was previously validated in a variety of cancers including medullary thyroid cancer (MTC), small cell lung, colon and ovarian cancers as well as in gliomas 4-7. Unfortunately, responses of these malignant tumors to conventional radio-chemotherapy are only transient and the benefit is limited to a small number of patients 8-12. Thus, any improvement of the standard-of-care cancer treatment would benefit a large number of patients. The agonist small peptide hormone minigastrin binds to CCKBR with high affinity and leads to receptor activation and internalization by endocytosis which involves clathrin-coated pits and dynamin as well as the binding of β-arrestin-1 or -2 13. Previous studies developed radiolabeled gastrin analogues with favorable pharmacokinetics and suggested them as attractive radiopharmaceuticals for theranostics (therapy and diagnostics) applications 14-19. The radiolabeled minigastrin [^111^In]In-labeled CP04 efficiently bound to cancer lesions in the CCKBR-positive *in vivo* tumor model 20, whereas clinical data obtained by SPECT imaging from MTC patient demonstrates high intratumoral accumulation of radiolabeled minigastrin [^177^Lu]Lu-PP-F11N 21. Furthermore, comparative biodistribution analysis in the mouse model shows that [^177^Lu]Lu-PP-F11N reached a tumor uptake that other Lu-117-labeled mingastrin analogues could only achieve in combination with protease inhibitors indicating its high metabolic stability 21. A more recent clinical study shows favorable biodistribution and pharmacokinetics of ^177^Lu[Lu]-PP-F11N and low kidney radiation doses with a median tumor-to-kidney dose ratio of 11.6 22. Nevertheless, in the latter study, radiolabeled minigastrin also accumulated in the stomach due to endogenous expression of the CCKBR and reached a tumor-to-stomach dose ratio of 3.34. Here, we demonstrate a clinically feasible way for augmented tumor-specific uptake of [^177^Lu]Lu-PP-F11N by the pharmacological interference with mTORC1 activity, which led to increased CCKBR protein levels in cancer cells, and consequently higher uptake of radiolabeled minigastrin.

## Material and Methods

### Cell culture, transfection and treatments

The human epidermoid carcinoma A431 cell line, which overexpresses CCKBR, was generated and kindly provided by Dr. Luigi Aloj 23. The A431 and A431/CCKBR cell lines were cultured in DMEM, whereas the rat pancreatic acinar AR42J cells (ECACC, UK) were grown in RPMI medium, supplemented with 10% FCS, 2 mM glutamine and antibiotics (0.1 mg/mL streptomycin, 100 IU penicillin) at 37 °C and 5% CO_2_. For CCKBR-specific knock-down, duplex siRNAs against CCKBR or control duplex against luciferase (Microsynth) were used at a final concentration of 100 nM in Optimem (Gibco): CCKBRseq1 sense RNA 5′-UAUACGAGUAGUAGCACCAdTdT-3′, CCKBRseq2 sense RNA 5′-CCGCCAAAGGAUGGAGUACdTdT-3′ and control sense RNA 5′-CGUACGCGGAAUACUUCGAdTdT-3′. Cells at 60-80% confluence were transfected with siRNAs by using Lipofectamin 3000 according to the manufacturer's recommendations. RAD001 (Selleckchem), rapamycin (Enzo Life Sciences), BML-257(Enzo), SC-514 (Enzo) or metformin (Selleckchem) were diluted in DMSO or water, respectively.

### Radiolabeling

The minigastrin analogue PP-F11N (DOTA-(DGlu)_6_-Ala-Tyr-Gly-Trp-Nle-Asp-Phe-NH2) was obtained from PSL GmbH, whereas lutetium-177 chloride solution ([^177^Lu]Lu) from ITG GmbH. The labeling reaction contained 1:30 nuclide/peptide ratio in 0.4 M ammonium acetate buffer (pH 5.5) and was carried out at 90 °C for 15 min. ^177^Lu incorporation was analyzed by HPLC using a C18 column and reached above 95% efficiency. For SPECT imaging, unlabeled PP-F11N was separated from the radiolabeled PP-F11N using a Merck Hitachi LaChrom 2D HPLC system, equipped with a D-7000 interface, a L-7200 auto sampler, a radiation monitor (RM-19, EBERLINE Instrument Corporation), a UV detector (Pharmacia LKB-UV-M II), a 515 HPLC pump and a L-7100 pump connected with a reversed-phase C18 cartilage and column. Elution was done using H2O:0.1% TFA (A) and ACN:0.1% TFA (B) linear gradients with 32-90 % B over the course of 30 min at a flow rate of 1 mL/min. Collected and speed-vac concentrated radioactive fractions were diluted in PBS and used for *in vivo* study.

### Kinase inhibitor library screen and proliferation assay

The Screen-well® Kinase Inhibitor Library comprising 80 inhibitors (Table S1) diluted in DMSO (10 mM) was from Enzo Life Sciences AG. 25.000 cells per well on isoplate 96 TC (PerkinElmer) were subjected to treatment with 10 μM kinase inhibitors for 18 h. On the next day, 0.03 pmol of radiolabeled [^177^Lu]Lu-PP-F11N (10.000 cpm) was added to each well and the plate was incubated for 2 h. The blocking control was performed with 4 µM of minigastrin LEEEEEAYGWMDF (PSL GmbH). After the removal of the radioactive supernatant, cells were resuspended in 50 µl of ULTIMA GOLD high flash-point LSC-cocktail (Sigma) and incubated for 2 h at RT. The activity was measured using the MicroBeta 2450 Microplate Counter (PerkinElmer). Cell proliferation was analyzed by a CellTiter 96 AQueous Non-Radioactive Cell Proliferation Assay (Promega). Absorbance of MTS bio-reduced into a formazan was measured at 570 nm with a reference of 650 nm, using a MicroPlate Reader (PerkinElmer). All experiments (screen and proliferation) were assayed in triplicate.

### Cell uptake and internalization assay

2.5 × 10^5^ cells (per well) on 6-well plates were treated with inhibitors for 20 h. On the next day, the cells were incubated with 0.3 pmol of radiolabeled [^177^Lu]Lu-PP-F11N (100.000 cpm) in DMEM with 0.1% BSA at standard TC condition for 1 h. For blocking, 4 µM minigastrin (PSL GmbH) was added. Radioactive medium (and PBS wash) was collected and the cells were washed 2× with glycine buffer (pH=2) for 5 min at RT followed by a dissolving step in 1 M NaOH for 15 min at 37°C. All 3 fractions (medium/PBS; glycine; dissolved cells) were measured on Cobra II Auto-Gamma counter (Packard). For the cell uptake analysis, the PBS-washed cells were directly dissolved in 1M NaOH, or combined glycine-wash and 1M NaOH dissolved cells, were measured on the Gamma counter. Results from the experiments with the blocking peptides demonstrated activity below 0.5% of total activity (Figure S1A) and were subtracted from obtained results. Protein concentration of cell lysates was measured by using NanoPhotometer® UV-Vis P-Class (Implen).

### WB analysis

Antibodies against phospho-S6 at S235/S236 (D57.2.2F), ERK1/2 (9102), phospho-ERK1/2 (T202/Y204), GAPDH (14C10) and HA-Tag (C29F4) were obtained from Cell Signaling Technology, whereas the anti-CCKBR (ab77077) was from Abcam. Cells were homogenized in lysis buffer (50 mM Tris-HCl pH 7.5, 150 mM NaCl, 1% Triton X, 0.1% SDS supplemented with 1 mM sodium orthovanadate, 1 mM NaF and protease inhibitor cocktail (Roche)). 50 μg of total protein lysates were separated by SDS-PAGE and transferred to PVDF membranes (Millipore) by electroblotting. Membranes were blocked with 5% skim milk in TBST (0.1% Tween 20) for 1 h, and incubated with 2% BSA in TBST overnight with the primary antibody followed by 2 h incubation with HRP-conjugated secondary antibody. Signals were produced by using a chemiluminescence reagent (ECL) and acquired by ImageQuant RT ECL Imager (GE Healthcare). Prior to CCKBR detection by WB, protein lysates were subjected to deglycosylation. Briefly, 18 μL of total protein lysates (50 µg) were mixed with 2 μL of 10× denaturing buffer (5% SDS, 0.4 M DTT) and incubated for 10 min at RT. Next, 4 μL of 10× Glycobuffer (0.5 M sodium phosphate, pH 7.5), 4 μL of 10% Tween-20 and 10 μL of water were added. Finally, 2 μL of PNGase F (Sigma) was added and the reaction was carried out for 16 h at 37°C.

### Split Luciferase Assay

Cells at 70-90% confluence on the 6-well plate were transfected with 2 μg per well of each pSI-AGR10 plasmid 24, 25 with subcloned human 114-βArrestin-1, 114-βArrestin-2, 11S-CAAX and CCKBR by using Lipofectamine 3000 according to the manufacturer's recommendations. Twenty-four h after transfection, 100,000 cells per well were plated into a 96-well plate (Perkin Elmer) and the cells were treated with RAD001 for 20 h. On the next day, the medium was exchanged to DMEM w/o phenol red (Bioconcept), supplemented with 20mM HEPES pH 7.0, 1% furimazine and 19% LCS dilution buffer (Nano-Glo® Live Cell Assay System, Promega) and a white cover was attached to the transparent bottom of the 96-well plate. Cells were stimulated with 1 µM of PP-F11N and the luminescence was measured using a PHERAstar FSX (BMG Labtech). After the recruitment assay, Hoechst fluorescence was measured to control cell number. Briefly, the microplates were frozen at -80°C for 1 h and 100 µl of dH_2_O was added to each well followed by 1 h incubation at 37°C. Then, the plates were placed at -80°C until frozen and thawed to RT. Next, per each well 100 µL of aqueous Hoechst 33258 (Sigma) diluted to a final concentration of 5 ug/ml in TNE buffer (10 mM Tris, 2 M NaCl, 1 mM EDTA, pH 7.4) was added and the fluorescence was measured using optic filters centred on 360 and 460 nm for excitation and emission, respectively.

### Animal study

All experiments were performed in accordance with Swiss Animal Protection Laws. For tumor implantation, 5 × 10^6^ of A431/CCKBR cells in 0.1 mL of phosphate-buffered saline (PBS) containing 0.9% NaCl were injected subcutaneously (two tumors per animal) into *CD-1* female nude mice (Charles Rivers, Germany) anesthetized by isoflurane/oxygen inhalation. After 5 days, the animals were randomly distributed into experimental groups and the tumor size was measured non-invasively with a caliper and the average tumor volumes were estimated for each group. RAD001 (3 mg/kg), metformin (200 mg/kg) or PBS (control) were administered daily via intraperitoneal injection. RAD001 and metformin doses were selected based on the previous animal studies, which show anti-tumor activity and no toxicity in the nude mice 26, 27. In the biodistribution study, mice received 3 pmol of radiolabeled [^177^Lu]Lu-PP-F11N into the tail vein (150 kBq in 0.1 mL PBS), for blocking 60 nmol minigastrin was co-injected and 4 h later the mice were subjected to euthanasia with CO_2_. Dissected tumors and organs were weighed and measured using the gamma counter.

For SPECT/CT imaging, [^177^Lu]Lu-PP-F11N was purified by HPLC and 0.2 nmol of radiolabeled [^177^Lu]Lu-PP-F11N was diluted in PBS (10 MBq in 100 µl). Two hours after i.v. injection, mice were sacrificed and subjected to 10 min X-ray computed tomography (CT) followed by 5 h single-photon emission computed tomography (SPECT) using a multipinhole small-animal NanoSPECT/CT camera (Mediso Medical Imaging Systems). Image reconstruction and quantification was accomplished by using connecting thresholding and Otsu method (VivoQuant 3.0 Patch1).

### Immunohistochemistry

Formalin-fixed, paraffin tumor sections were deparaffinized, rehydrated and pretreated in 10 mM citrate buffer, pH 6.0, at 98 °C for 60 minutes, followed by incubation with 4% fat-free milk in PBS for 90 minutes. For avidin/biotin blocker treatment (Invitrogen) and detection, the ABC method was used. For monoclonal antibody against Ki67 (SP6, Thermo Scientific) and CD34 (cloneQBend/10; Dako) signals were recorded using an automated instrument reagent system (Discovery XT, Ventana Medical System Inc.). Images of hematoxylin-counterstained sections were captured (Nikon, YTHM) and analyzed using ImageAccess Enterprise7 and ImageJ software 28. Tumor necrotic areas and vascularization were analyzed in 10 (RAD001 and PBS) and 8 (metformin) images, whereas Ki67 and mitotic index in 15 (RAD001 and PBS) and 12 (metformin) images.

### Statistics

Two-tailed Student's *t* tests were performed for analysis of two groups, whereas one-way ANOVA followed by multiple comparison tests were performed for three or more groups (GraphPad Prism 7.00). Values of *P*<0.05 were considered statistically significant.

## Results

### Inhibition of mTORC1 increases the internalization of [^177^Lu]Lu-PP-F11N

**Figure 1:**
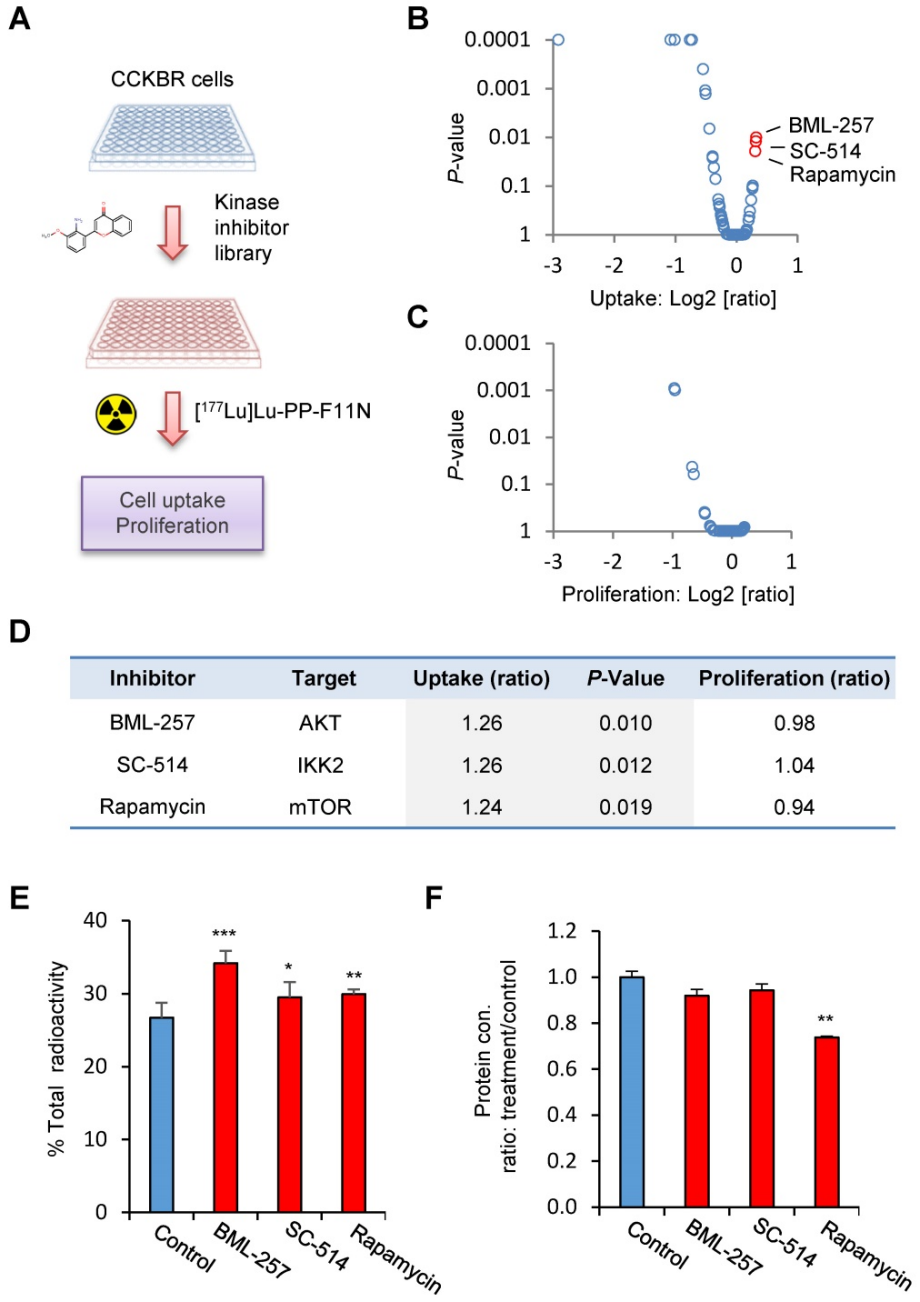
** Identification of kinase inhibitors for the enhancement of [^177^Lu]Lu-PP-F11N cellular uptake.** (**A**) Experimental design: Kinase inhibitor-treated and control untreated A431/CCKBR cells were subjected to the analysis of [^117^Lu]Lu-PP-F11N uptake. (**B** and **C**) Changes in [^117^Lu]Lu-PP-F11N cellular uptake and proliferation shown as log2 [ratio: treatment/control], respectively. Red dots represent inhibitors which significantly increased uptake of [^117^Lu]Lu-PP-F11N (*P*<0.05). (**D**) Identified inhibitors and their targets. Uptake and proliferation rates shown as ratios: treatment/control. (**E**) Cellular uptake of radioactivity after 1 h incubation with [^117^Lu]Lu-PP-F11N in control and 10 µM BML-257, SC-514 and Rapamycin-treated A431/CCKBR cells. Bars represent mean ± SD. (**F**) Mean protein concentration ± SD of lysates used in E. Mean protein concertation in the control cells was set to 1. *P<0.05, **P<0.01, ***P<0.001.

**Figure 2:**
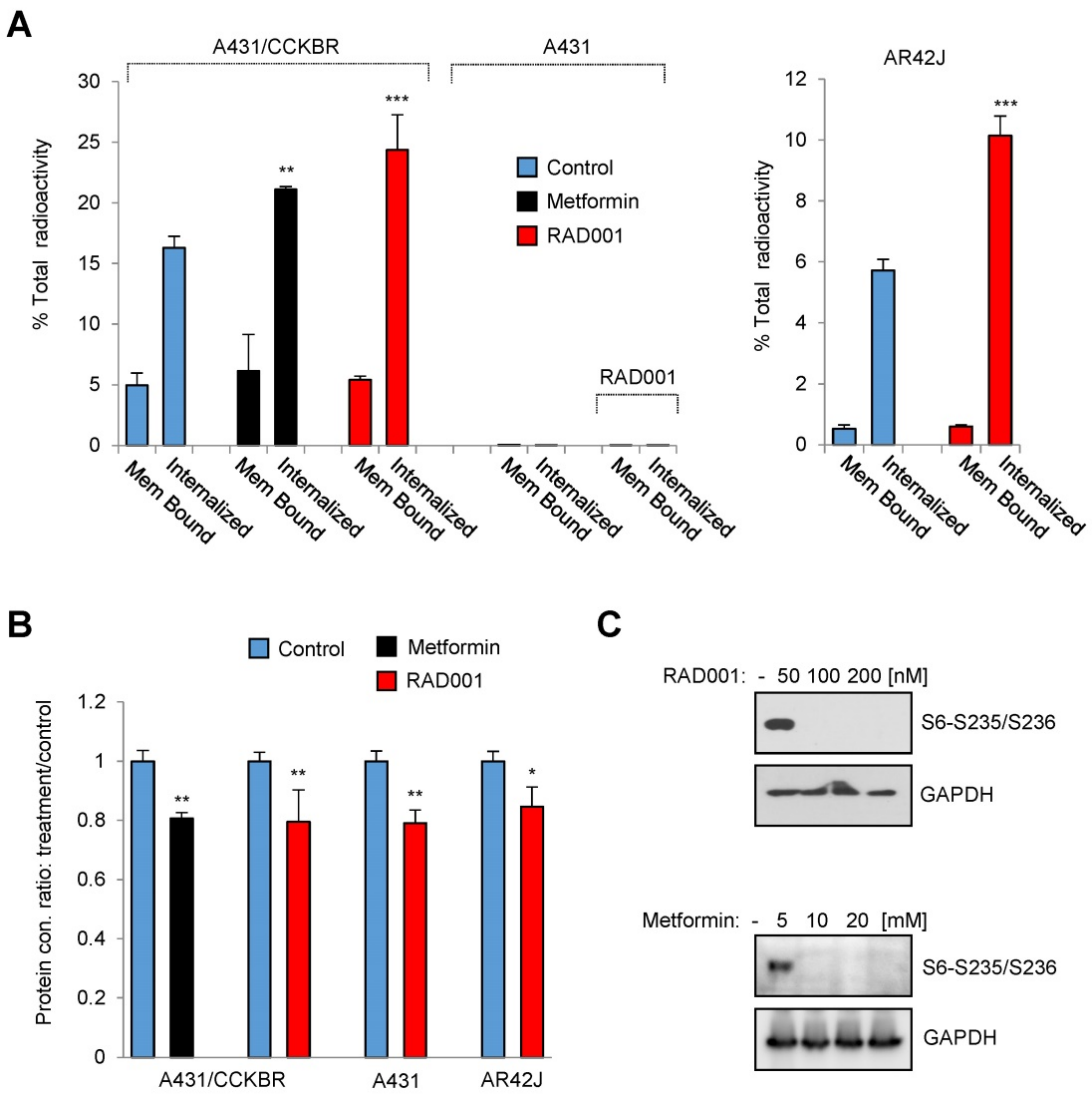
** Inhibition of mTORC1 activity increases internalization of [^177^Lu]Lu-PP-F11N.** (**A**) Internalized and cell-bound activity of [^177^Lu]Lu-PP-F11N in untreated control and 100 nM RAD001 or 10 mM metformin-treated A431/CCKBR, A431 and AR42J cells. All experiments were assayed in triplicate. Bars represent mean ± SD. (**B**) Mean protein concentration ± SD of lysates used in A. Mean protein concertation in the control cells were set to 1. (**C**) WB analysis using phosho-S6 antibody in RAD001- and metformin-treated A431/CCKBR cells. Blots were re-probed with GAPDH antibody for loading control. *P<0.05, **P<0.01, ***P<0.001.

In order to identify potential pathways which influence the internalization rate of the radiolabeled minigastrin analogue, we employed a kinase inhibitor library containing 80 inhibitors to analyze [^177^Lu]Lu-PP-F11N uptake in A431/CCKBR cells (Figure 1A). As expected, several inhibitors reduced uptake of [^177^Lu]Lu-PP-F11N as a result of inhibition of cell proliferation or viability. On the other hand, the screen identified 3 kinase inhibitors, BML257, SC-514, and rapamycin, which significantly enhanced [^177^Lu]Lu-PP-F11N cell uptake by 24-26% as compared to untreated control cells (Figure 1B and Table S1). Matching proliferation assays did not show significant changes in the cell proliferation for these inhibitors, indicating that the enhanced uptake did not result from the increased cell numbers after the treatment (Figure 1C-D). Validation experiments confirmed increased cellular uptake of [^177^Lu]Lu-PP-F11N in BML257, SC-514 and rapamycin-treated cells by 27-11%, whereby rapamycin treatment significantly decreased total protein level by 26% (Figure 1E-F). Two out of three identified inhibitors, BML-257 and rapamycin, target the AKT/mTORC1 pathway. In addition, rapamycin, a macrolide antibiotic, which inhibits mTOR kinase complex 1 (mTORC1), reduced protein level in treated cells indicating anti-cancer activity. Therefore, to further investigate the pharmacological interference with AKT/mTORC1 signaling, we took advantage of the clinically approved mTORC1 inhibitors; RAD001 (Everolimus) and metformin. Twenty hours metformin or RAD001 treatment of A431/CCKBR cells significantly increased [^177^Lu]Lu-PP-F11N internalization to 21.1 or 24.3%, respectively, as compared to 16.2% in control cells (ratio [treatment/control] of 1.3 and 1.5), whereas RAD001-treatment in AR42J cells showed a significantly increased internalization of 10% as compared to 5.7% in the control cells with the ratio [RAD001/control] of 1.7. There was no significant difference in the membrane-bound activity in RAD001 or metformin treated cells (Figure 2A). RAD001-treated and untreated A431 cells, which do not express CCKBR, did not show internalization of [^177^Lu]Lu-PP-F11N. Total protein levels in RAD001- and metformin-treated A431/CCKBR cells as well as RAD001-treated A431 and AR42J cells were significantly reduced to 79, 80 and 79, 84%, respectively, as compared to control (Figure 2B). The effects of RAD001 and metformin treatments were examined by WB analysis and showed lack of mTORC1-regulated phosphorylation of the ribosomal protein S6 at Ser235/236 in protein lysates of treated cells (Figure 2C). Similarly, 20 h incubation with 50, 100 and 200 nM RAD001 increased uptake of [^177^Lu]Lu-PP-F11N and inhibited S6 phosphorylation in A431/CCKBR cells (Figure S1B).

### RAD001 treatment increases CCKBR protein level

**Figure 3:**
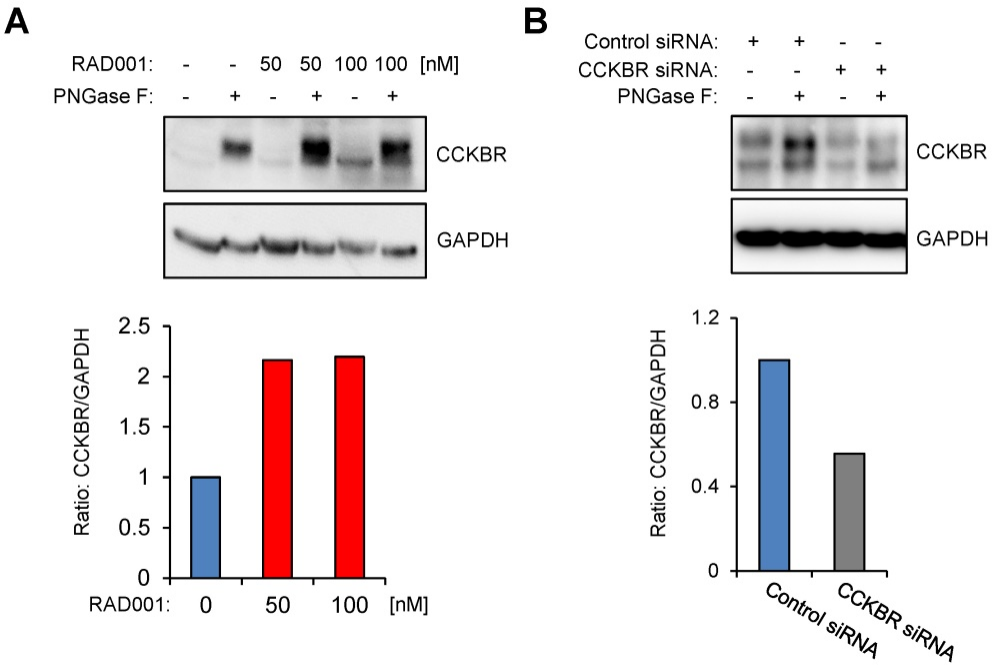
** RAD001 treatment increases CCKBR protein level.** WB analysis using anti-CCKBR antibody of the glycosylated and deglycosylated (PNGase F-treated) lysates from A431/CCKBR cells, following 20 h RAD001 treatment (**A**) or transfection (**B**) with luciferase siRNA (control) and CCKBR siRNA for 48 h, as indicated. Blots were re-probed with GAPDH antibody. Below; quantification of the CCKBR signal intensities in deglycosylated lysates normalized to GAPDH. The CCKBR/GAPDH ratios from untreated or control transfected cells were set to 1.

**Figure 4:**
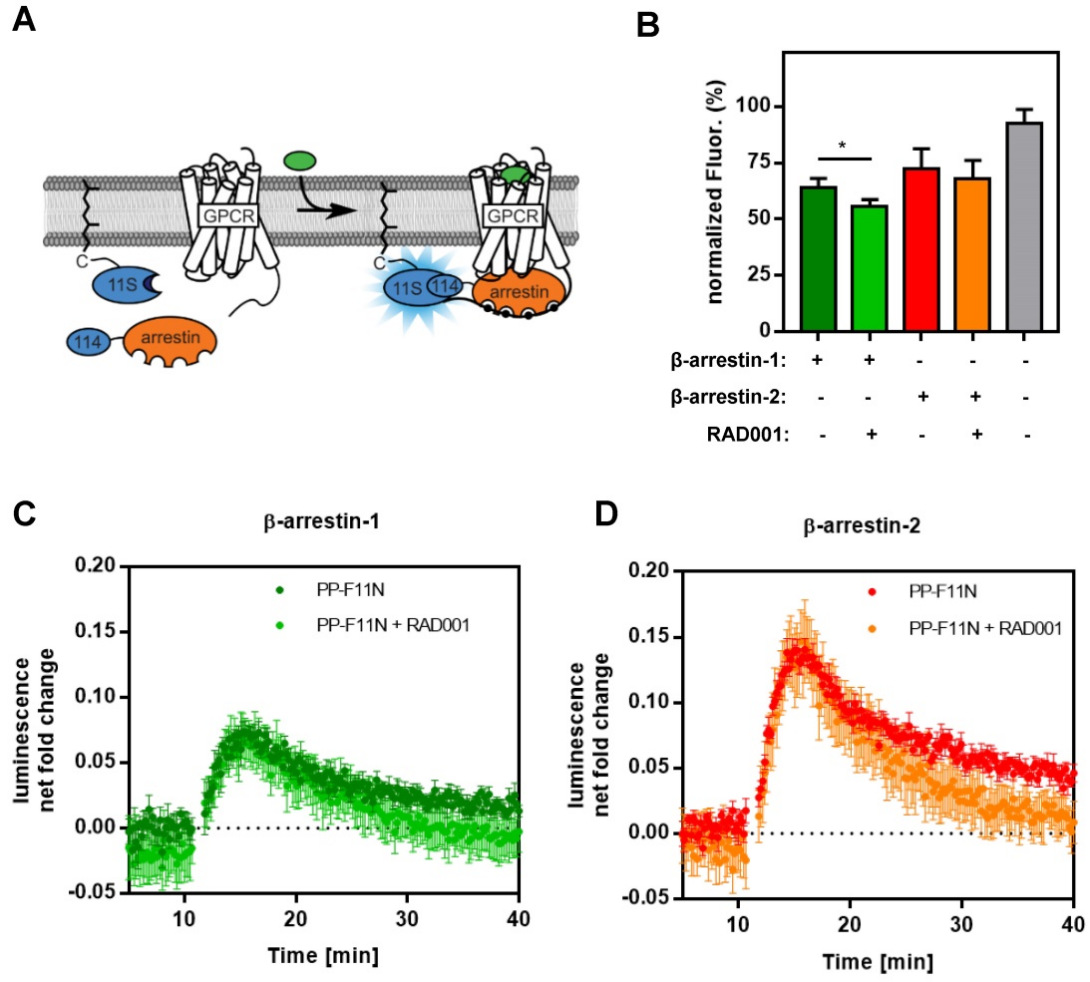
** Recruitment of β-arrestin-1/2 in PP-F11N-stimulated A431/CCKBR cells.** (**A**) Principle of the assay. The large subunit of NanoLuc is expressed with a prenylation signal (11S-CAAX) localizing it to the plasma membrane. The small subunit (114) is linked to the N-terminus of arrestin. These components are transiently transfected in human cells. GPCRs are expressed untagged. Upon stimulation, arrestin is recruited to the GPCR at the plasma membrane and chemiluminescence is enhanced. (**B**) Hoechst staining indicates relative cell amount used in arrestin recruitment assay performed in triplicate. The maximum fluorescence was set to 100 %. Data represent mean of normalized fluorescence ± SD. (**C** and **D**) Graphs represent the recruitment of human β-arrestin-1 and β-arrestin-2 to CCKBR in cells either treated with RAD001 or untreated, both conditions were stimulated with 1 µM of PPF-11N. Collected data was divided by the baseline luminescence, yielding the luminescence net fold change after treatment and are shown as mean ± SD. Time courses of cells not treated with PP-F11N were subtracted. *P<0.05.

To investigate CCKBR protein level in response to RAD001 treatment, we first established WB protocols by using an anti-HA antibody on protein lysates isolated from U-251MG/CCKBR-HA cells, which overexpress HA-CCKBR and internalize [^177^Lu]Lu-PP-F11N. As shown in Supplementary Figure S2, the specific detection of CCKBR required a deglycosylation step prior to WB analysis, whereby we observed a signal shift from 100-76 kDa to approximately 48-46 kDa after deglycosylation. Furthermore, CCKBR was detected only in the deglycosylated A431/CCKBR cell lysate by using a polyclonal anti-CCKBR antibody. Next, the expression of CCKBR in A431/CCKBR cells was further studied in RAD001-treated cells. As shown in Figure 3A, treatment with either 50 or 100 nM RAD001 increased CCKBR levels (2.2-fold), indicating that increased internalization of the radiolabeled minigastrin in RAD001-treated cells resulted from elevated CCKBR levels. The specific detection of the CCKBR was verified by RNA interference, whereby transfection with the duplex CCKBR siRNAs reduced the CCKBR expression level to 55 % as compared to control cells transfected with duplex siRNAs against the luciferase gene (Figure 3B). In our study, the internalization of the radiolabeled minigastrin analogue suggests that PP-F11N acts as an agonist. Furthermore, stimulation of A431/CCKBR cells with PP-F11N increased phosphorylation of extracellular signal-regulated kinase 1 and 2 (ERK1/2) as shown by WB analysis in Figure S3, indicating CCKBR effect for an agonist ligand. To investigate this point further, we analyzed recruitment of the β-arrestin upon stimulation with an agonist peptide PP-F11N in RAD001-treated cells, by employing a split luciferase assay in β-arrestin-1 or -2 and CCKBR transfected cells (Figure 4A). Similarly to the previous experiments, RAD001 pretreatment significantly reduced cell number as determined by Hoechst fluorescence (Figure 4B). Stimulation with PP-F11N triggered recruitment of both β-arrestins to the CCKBR, whereby β-arrestin-2 showed a stronger interaction (Figure 4C-D). There was no difference in arrestin recruitment between cells treated with RAD001 and untreated cells.

### Enhanced uptake of [^177^Lu]Lu-PP-F11N in RAD001-treated CCKBR tumors

**Figure 5:**
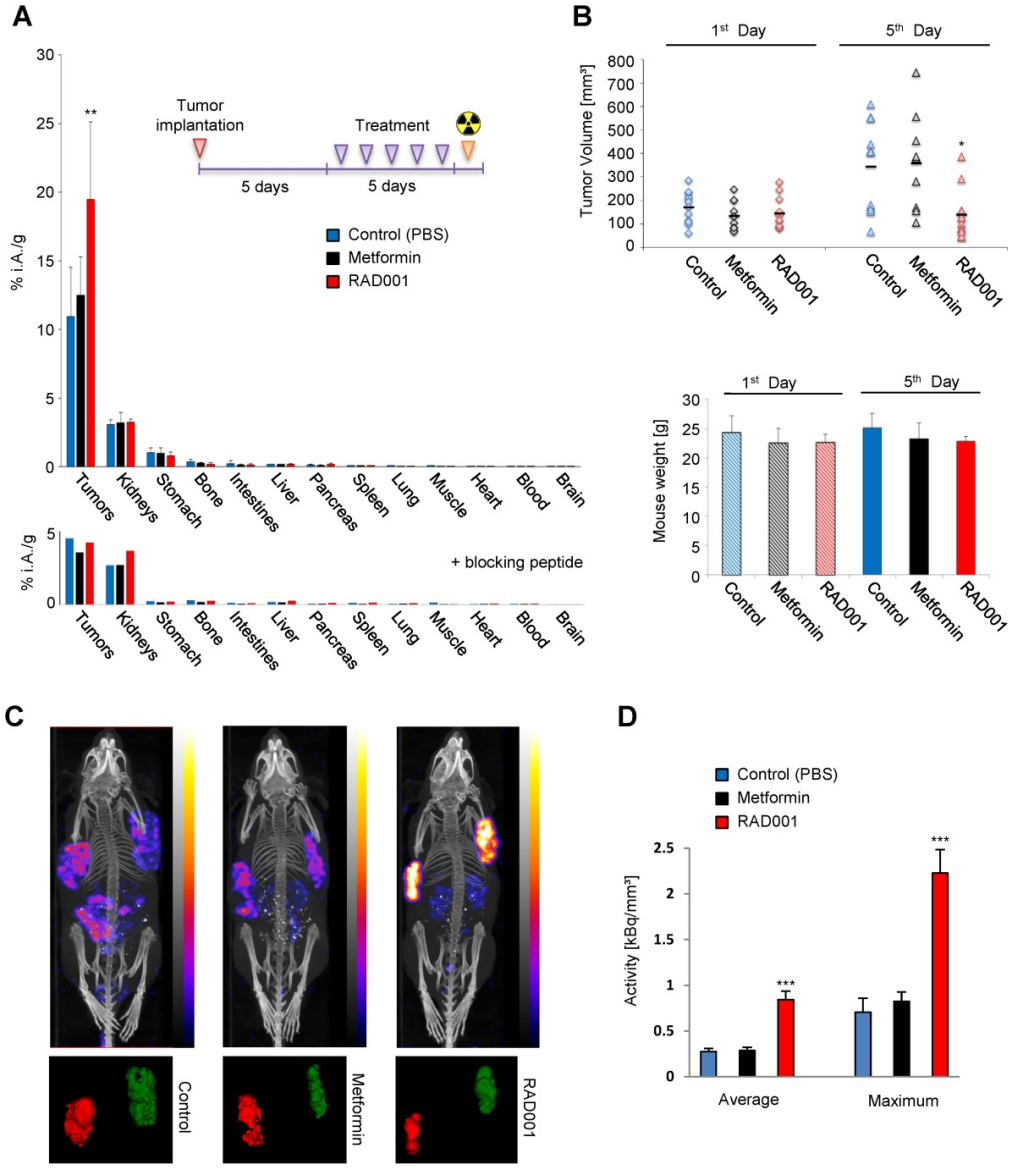
** RAD001 enhances CCKBR-dependent tumor uptake of [^177^Lu]Lu-PP-F11N.** (**A**) After implantation of A431/CCKBR cells into nude mice, RAD001 (n=6), metformin (n=5) and PBS (n=6) groups were treated daily for 5 days. Bars; biodistribution of [^177^Lu]Lu-PP-F11N shown as % of total injected radioactivity per gram of tissue (% i.A./g). Lower panel; Corresponding biodistribution after co-injection with a blocking peptide in one mouse per group. (**B**) Tumor volume and mouse weight before (1^st^ day) and after treatment (5^th^ day). Bars represent mean ± SD. (**C**) SPECT/CT images after [^177^Lu]Lu-PP-F11N injections in RAD001, metformin, and control (PBS) treated mice. Below: corresponding radioactive regions in left (red) and right (green) flank tumors. (**D**) Average and maximum activity concentration ± SD of [^177^Lu]Lu-PP-F11N in radioactive regions of 4 tumors per group. *P<0.05, **P<0.01, ***P<0.001.

Biodistribution analysis in A431/CCKBR tumor-bearing mice demonstrates significant increase (*P*=0.0057) in the tumor uptake of [^177^Lu]Lu-PP-F11N in RAD001-treated animals with a ratio [RAD001/Control] of 1.79, whereas metformin treatment increased [^177^Lu]Lu-PP-F11N uptake only moderately with a ratio [Metformin/Control] of 1.14 and did not reach statistical significance (Figure 5A). There was no significant difference in biodistribution of [^177^Lu]Lu-PP-F11N in the healthy organs in all groups, including the stomach which expresses CCKBR (Table S2). Co-injection of [^177^Lu]Lu-PP-F11N with a blocking peptide reduced [^177^Lu]Lu-PP-F11N uptake in the tumor and stomach (Figure 5A lower panel). The kidney uptake was not affected by the blocking peptide due to the general mechanism of glomerular filtration of the peptides in the primary urine. RAD001 significantly reduced tumor volumes without influencing overall mouse weight (Figure 5B). Metformin had no effects on either the tumor volume or mouse weight. During treatment cycles, no signs of acute toxicity were observed in any of the groups. In order to investigate if the increased tumor uptake was influenced by the difference in the tumor masses between RAD001 and the control group, we performed statistical analysis in the tumor groups with matching sizes. RAD001-treated tumors ranging from 50-150 or 50-180 mg showed statistically significant increase in [^177^Lu]Lu-PP-F11N uptake without any significant differences in the tumor masses, indicating that RAD001 treatment increases tumor uptake of [^177^Lu]Lu-PP-F11N in a tumor mass-independent manner (Figure S4). Next, we analyzed if less than 5 doses of RAD001 would increase [^177^Lu]Lu-PP-F11N tumor uptake. Biodistribution analysis showed a significant (*P*=0.016) increase in the tumor uptake of [^177^Lu]Lu-PP-F11N in mice treated with 3 daily RAD001 doses with ratio [RAD001/Control] of 1.56 (Figure S5A). Three doses of metformin or one RAD001 dose influenced [^177^Lu]Lu-PP-F11N uptake only moderately and did not reach statistical significance (Figure S5B-C). There was no significant difference in the biodistribution of [^177^Lu]Lu-PP-F11N in healthy organs in all groups. Three daily doses of RAD001 significantly reduced tumor volumes without influencing overall mouse weight, whereas metformin treatment had no effects on either tumor volume or mouse weight (Figure S5D). There was a linear correlation (R² = 0.96) between the number of administrated doses and tumor uptake (Figure S6). Notably, after 1, 3 and 5 daily doses of RAD001, the activity level in the blood remained unchanged and was very low (below 0.05 % of total injected activity, 4h post injection), which suggests that RAD001 treatment does not affect the pharmacokinetics of [^177^Lu]Lu-PP-F11N.

SPECT/CT imaging confirmed increased tumor uptake of [^177^Lu]Lu-PP-F11N in the RAD001-treated A431/CCKBR-tumor bearing mice (Figure 5C), whereby relative quantification demonstrated significantly higher (*P*<0.001) average and maximum concentration of [^177^Lu]Lu-PP-F11N in RAD001-treated tumors (Figure 5D). The ratios [RAD001/Control] of the average and maximum concentration reached 3.11 and 3.17, respectively, whereas metformin treatment did not show a significant change (with the average and maximum concentration ratios [Metformin/Control] of 1.03 and 1.17, respectively).

### Increased necrosis and reduced number of the mitotic figures and Ki67 positive cells in RAD001-treated tumors

**Figure 6:**
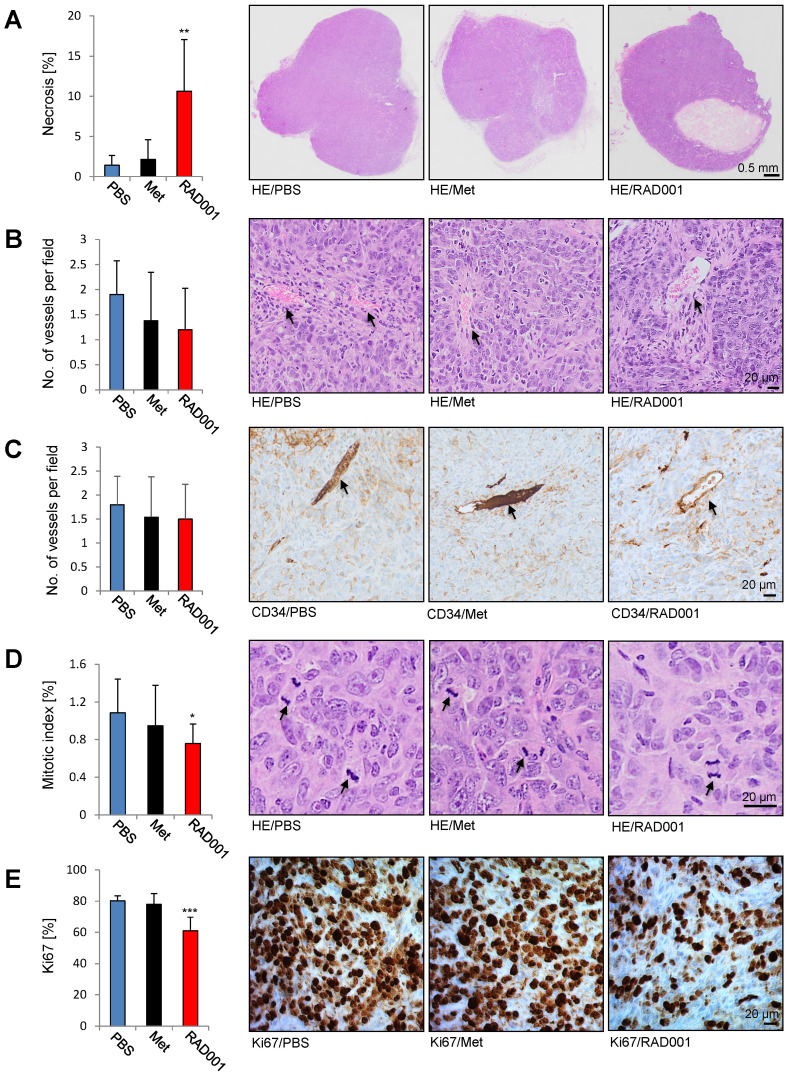
** Increased necrosis and reduced number of the mitotic figures and Ki67 positive cells in RAD001-treated tumors.** Paraffin sections prepared from A431/CCKBR tumors treated with RAD001, Metformin (Met) and PBS (control) were subjected to HE, CD34 and Ki67 staining. Bars represent percent of necrotic area (**A**), no. of vessels per field (**B** and **C**), mitotic index (**D**) and percent of Ki67 positive cells (**E**) shown as an average ±SD of analyzed tumor groups. Right; images from representative HE and Ki67 staining. Arrows in B, C indicate vessels, and in D mitotic figures. Scale bar 0.5 mm in A and 20 µm in B-D. *P<0.05, **P<0.01, ***P<0.001.

Paraffin A431/CCKBR-tumor sections prepared after RAD001, metformin and PBS (control) treatment were subjected to hematoxylin and eosin (HE) stain and immunohistochemistry (IHC). Each RAD001 and PBS group included 5 tumors, while in the metformin group 4 tumors were analyzed. As shown in Figure 6A, RAD001 increased necrosis (10.6 ± 6.4%) as compared to control PBS-treated tumors (1.4 ± 1.2%), whereas metformin did not show a significant difference. Analysis of the vessel numbers by HE staining and CD34 immunohistochemistry did not show any statistical difference between the analyzed groups indicating that increased radiolabeled* in vivo* minigastrin uptake is due to the increased CCKBR protein levels in RAD001-pretreated tumors (Figure 6B-C). A significantly reduced mitotic index (0.75 ± 0.36%) and number of Ki67 positive cells (61 ± 8.6%) was detected in RAD001-treated tumors as compared to control tumors (1.08 ± 0.2% of mitotic index and 80 ±3.1% Ki67 positive cells). Metformin treatment did not cause a significant change in the number of mitotic figures or Ki67 positive cells (Figure 6D-E).

## Discussion

One of the most important properties for the efficient PRRT is a high ratio of tumor-to-normal tissue uptake of the radioactive compound, which possesses high selectivity and affinity towards the target receptor. In addition to the compound affinity or stability, tumor uptake largely depends on the expression level of the targeted receptor. Currently, kinase inhibitors represent one of the largest classes of approved drugs for targeted cancer therapies 29. Many previous studies employed kinase inhibitor library screens to investigate molecular mechanisms underlying carcinogenesis and therapy resistance, as well as to identify novel drug targets 30. Thus, in the present study we screened a kinase inhibitor library to identify compounds which can enhance uptake of the radiolabeled minigastrin in CCKBR-expressing cells. Two out of three identified inhibitors target the oncogenic AKT/mTOR pathway that supports cancer cell growth and survival 31. In order to develop a new therapeutic approach for use in the clinic we employed the allosteric mTORC1 inhibitor RAD001 (Everolimus), previously approved for the treatment of advanced renal cell carcinoma and subependymal giant cell astrocytoma, as well as the diabetes-approved drug metformin, which inhibits mTORC1 indirectly via activation of AMP-activated protein kinase (AMPK) 32-34. In our study, pharmacological inhibition of mTORC1 increased the level of CCKBR, suggesting that interfering with the growth pathways may effect CCKBR expression or stability. Indeed, previous study in pancreatic rat cancer AR42J cells showed increased CCKBR expression during starvation where the cellular growth is inhibited and associated with an inactivation of the mTORC1 pathway 35. Consistently, in our study, inhibition of mTORC1 increased CCKBR-specific internalization of the radiolabeled minigastrin in rat AR42J as well as in human A431/CCKBR cells. AR42J cells express endogenous CCKBR, whereas stable transfected A431 cells express CCKBR ectopically 23. Thus, the study data suggests that RAD001-mediated increase in CCKBR level and the internalization rate results from increased stability of the CCKBR in RAD001-treated cells and represents a general mechanism which can be applied to various models. On the one hand, inhibition of mTORC1 by rapalogs, including RAD001, reduces general protein synthesis but on the other hand, it triggers selective synthesis of stress-related proteins or oncogenes 36. Activated mTORC1 can also directly phosphorylate certain proteins and facilitate their degradation. For example, in response to growth factors mTOR-mediated phosphorylation of dual specificity phosphatase 6 (DUSP6) at serine 159 leads to its degradation 37. In addition, inhibition of mTORC1 can influence protein stability by regulating enzymes involved in posttranslational modifications such as O-GlcNAcylation 38. Thus, targeting mTORC1 by RAD001 can directly influence protein stability. Nevertheless, since a previous study reported increased CCKBR mRNA expression in AR42J cells during starvation 35, the transcriptional regulation of CCKBR expression in RAD001-treated cells cannot be excluded.

In addition to increased CCKBR protein levels in RAD001-treated cells, we analyzed β-arrestin-1/2 recruitment and did not observe any difference. Although arrestin is responsible for initiating the internalization of many GPCRs, the internalization rate does not always equal arrestin recruitment and the internalization of some GPCRs is independent of arrestin 39-42. Alternative internalization mechanisms apart from the clathrin-coated pits initiated by arrestin are known, including caveolin- and flotillin-mediated internalization 43. Notable, previous study data shows arrestin recruitment in natural ligand CCK or gastrin-stimulated cells but also demonstrates that β-arrestin-1/2 are dispensable for the internalization of CCKBR, whereby internalization of CCKBR was abundant immediately after CCK stimulation in β-arrestin1/2 double knock-out MEF cells 13. This result supports the existence of an arrestin-independent mechanism for CCKBR internalization, which could be activated in response to mTORC1 inhibition or its downstream signaling. Further clarification of molecular mechanisms involved in the regulation of CCKBR protein expression and the PP-F11N internalization in RAD001-treated cells warrants additional investigation.

Glycosylation on the N-terminus of GPCRs is involved in the localization of the receptor on the plasma membrane. Given that there are 3 known N-terminus glycosylation sites on the human CCKBR receptor (R7, R30 and R36) 44 we found that CCKBR exists in a glycosylated form and that a deglycosylation step is necessary for the assessment of endogenous CCKBR protein levels. This suggests that the glycosylation contributes to the epitope masking. Thus, our data strongly recommend a deglycosylation step prior to detection of CCKBR by WB, at least by using the antibody tested in our study.

Metformin is a less potent inhibitor than RAD001 and it inhibits mTORC1 indirectly via activation of AMPK, which in turn phosphorylates mTORC1 negative regulator, the tuberous sclerosis complex (TSC) 1/2 45. The differences in response to rapamycin and metformin treatment have been previously demonstrated. For example, rapamycin but not metformin decreased expression of vascular endothelial growth factor (VEGF) in hepatocellular carcinoma cells 46. Furthermore, rapamycin treatment inhibited the growth of hepatocarcinoma tumors inoculated in NOD-SCID mice to a greater extent as compared to metformin 47. Similarly in our study, 5-day metformin treatment did not affect tumor growth and [^177^Lu]Lu-PP-F11N uptake in contrast to RAD001 treatment. This suggests different effects of metformin and RAD001 on the regulation of gene expression *in vivo*. We can also not exclude insufficient anti-tumor activity of metformin in our animal model. Thus, the use of metformin to increase uptake of [^177^Lu]Lu-PP-F11N *in vivo* needs further investigation.

In our study we employed the human A431/CCKBR xenograft mouse model. This model was broadly used for radiolabeled gastrin analogue development and the preclinical evaluation of pharmacokinetics, biodistribution, dosimetry or toxicity, required for regulatory approval of a phase I clinical trial with MTC patients 20. In the RAD001-treated A431/CCKBR-tumor mouse model, we observed enhanced CCKBR-specific uptake of the radiolabeled minigastrin in tumors, but not in the healthy organs such as the gastrointestinal tract which expresses endogenous CCKBR. This might be explained by the fact that, in contrast to healthy tissue, cancer cells have high mTORC1activity. Notably, MTC results most often from sporadic mutations, frequently affecting the proto-oncogene tyrosine-protein kinase receptor RET (rearranged during transfection) 48. Mutated and constitutively active RET regulates protein kinase AKT, which in turn, activates mTORC1, a major growth regulator. Activation of the mTOR pathway was previously demonstrated in human primary MTC and lymph node metastasis, whereby phosphorylation of S6, a downstream target of mTORC1, was evident in 96% of MTC samples 49. The study also showed blocked viability and motility of rapamycin or RAD001-treated MTC cells. A more recent clinical study in patients with advanced MTC indicated that everolimus (RAD001) exerts clinically relevant antitumor activity at relatively low toxicity 50. In agreement with the antitumor activity of RAD001, we observed inhibition of tumor growth, increased necrotic areas as well as reduced cell proliferation in RAD001-treated tumors. Furthermore, the radiosensitizing effect of RAD001 was previously demonstrated in different cancer models including Ras-transformed cells or renal cell carcinoma, whereby RAD001-mediated radiosensitization was partially dependent on the induction of autophagy 51, 52. Thus, the RAD001 treatment can not only increase uptake of [^177^Lu]Lu-PP-F11N but it may also sensitize cancer cells to radiotherapy. Moreover, information on the toxicity of RAD001 in combination with a ^177^Lu-labeled DOTATATE was collected in a phase I study and demonstrated that a daily intake up to 7.5 mg of RAD001 for 24 weeks pertains to the maximal tolerable dose and indicates that use of RAD001 in combination with PRRT is clinically applicable 53.

Notably, a previous study also suggested improvement of the tumor uptake by combinatory treatment, whereby co-administration of the neutral endopeptidase inhibitor phosphoramidon (PA) increased the level of circulating radiopeptides, including the gastrin analogue [^111^In]In-DOTA-MG11, and remarkably enhanced tumor uptake in mouse models 54. In contrast to our method, this combinatory strategy robustly increased uptake in the stomach, a dose-limiting organ for PRRT with radiolabeled minigastrin 22, and did not affect tumor viability or radio-sensitivity, which additionally can increase therapeutic responses. In addition, mTORC1 inhibition can normalize tumor vessels and enhance delivery of chemotherapeutics such as paclitaxel as previously demonstrated in rapamycin-treated breast cancer mouse models 55. This suggests that the observed enhanced tumor uptake of radiolabeled mingastrin *in vivo* might also result from the normalized tumor vascularization after RAD001 treatment. However, we observed no significant differences in the tumor vascularization after 5 days of RAD001 treatment, which might be explained by the relatively short treatment duration. In addition, increased internalization rates of radiolabeled minigastrin in RAD001-incubated *in vitro* cultured cells further point to the CCKBR-dependent molecular mechanisms.

In conclusion, as revealed by the present study, enhanced tumor-specific uptake of [^177^Lu]Lu-PP-F11N due to increased CCKBR level in response to mTORC1 inhibition by RAD001, has the potential to substantially improve efficacy of PRRT and nuclear imaging of radiolabeled minigastrin analogues. Furthermore, the study recommends the development of molecular strategies aimed at enhancing expression or stability of the targeted receptors by using clinically approved cancer drugs.

## Supplementary Material

Supplementary figures and tables.

## Abbreviations

CCKBR: Cholecystokinin B receptor
GPCR: G-protein coupled receptor
PRRT: Peptide receptor radionuclide therapy
SPECT: Single-photon emission computed tomography
mTORC1: Mammalian target of rapamycin in complex 1
MTC: Medullary thyroid cancer
