# Supplementary material for: Pharmacological inhibition of mTORC1 increases CCKBR-specific tumor uptake of radiolabeled minigastrin analogue [177Lu]Lu-PP-F11N
Source: Theranostics. 2020 Aug 29;10(24):10861–73. doi: 10.7150/thno.45440 (PMC7532663; doi:10.7150/thno.45440)
Supplement: Supplementary file 1 — Supplementary figures and tables. [file thnov10p10861s1.pdf]

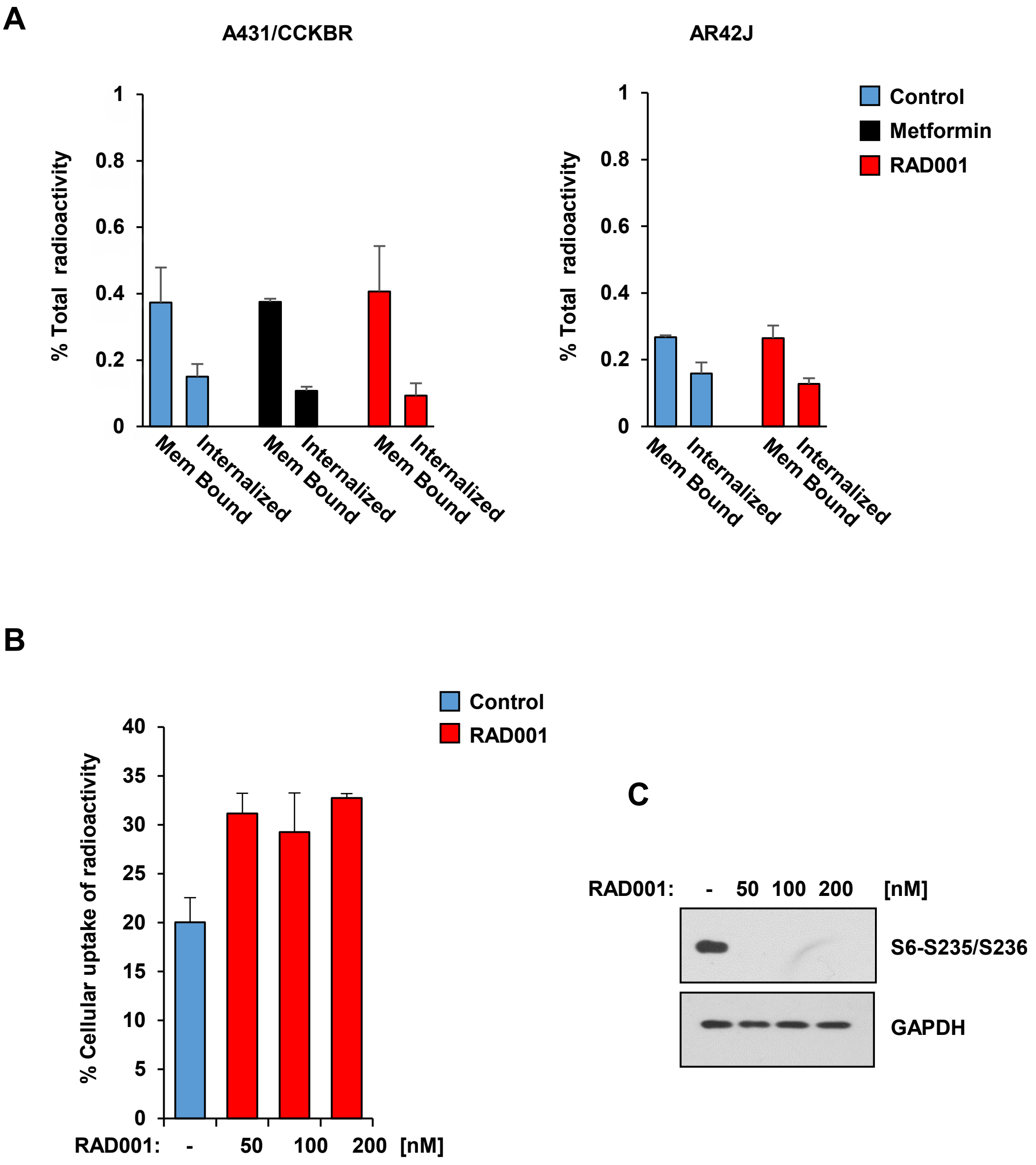


**Figure S1. Analysis of [$^{177}$Lu]Lu-PP-F11N cellular uptake in RAD001 and Metformin treated cells.** (**A**) Internalized and cell-bound activity of [$^{177}$Lu]Lu-PP-F11N in untreated control and 100 nM RAD001 and 10 mM metformin-treated A431/CCKBR and AR42J cells at the presence of a blocking peptide. All experiments were assayed in triplicate. Bars represent mean ± SD and correspond to the results shown in Figure 2. (**B**) Cellular uptake of radioactivity after 1 h incubation with [$^{177}$Lu]Lu-PP-F11N in control and 50, 100 and 200 nM RAD001-treated A431/CCKBR cells. Bars represent mean ± SD. (**C**) WB analysis using phosho-S6 antibody in RAD001-treated A431/CCKBR cells. Blots were re-probed with GAPDH antibody for loading control.

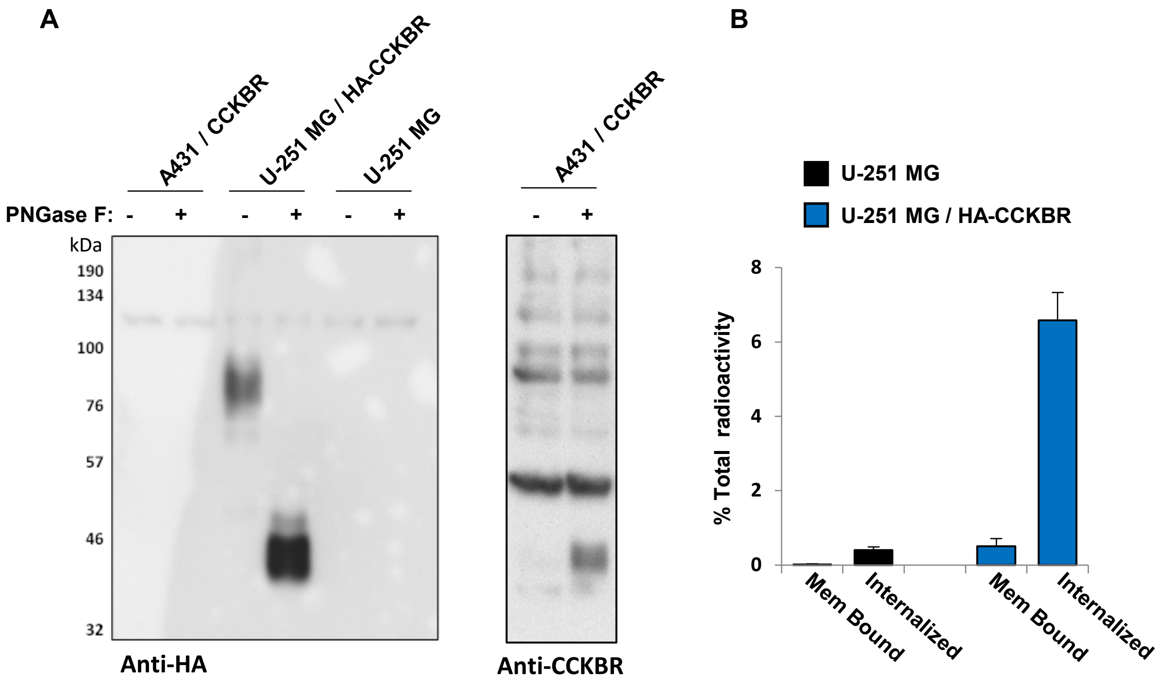


**Figure S2. Analysis of CCKBR expression and glycosylation.** (**A**) WB analysis using anti-HA or anti-CCKBR antibody of the glycosylated and deglycosylated (PNGase F-treated) total protein lysates isolated from A431/CCKBR as well as control untransfected and HA-CCKBR transfected U-251 MG cells. (**B**) Internalized and cell-bound activity was measured after 1 h incubation with [$^{177}$Lu]Lu-PP-F11N in control and HA-CCKBR transfected U-251 MG cells. All experiments were assayed in triplicate. Bars represent mean ± SD.

Glioma U-251 MG cells were from CLS, Germany and cultured in DMEM supplemented with 10% FCS, 2 mM glutamine and antibiotics (0.1 mg/mL streptomycin, 100 IU penicillin) at 37 °C and 5% CO2. For CCKBR overexpression in U-251 MG cells we used a pcDNA3.1+ construct with the human full length CCKBR HA-tagged at the N-terminus (from cDNA.org; catalog number: #CCKBR0TN00). Cells at 60-80% confluence were transfected using Lipofectamine 2000 (Invitrogen), and the stable cell line was generated and maintained by using 1 and 0.4 mg/ml of G418, respectively.

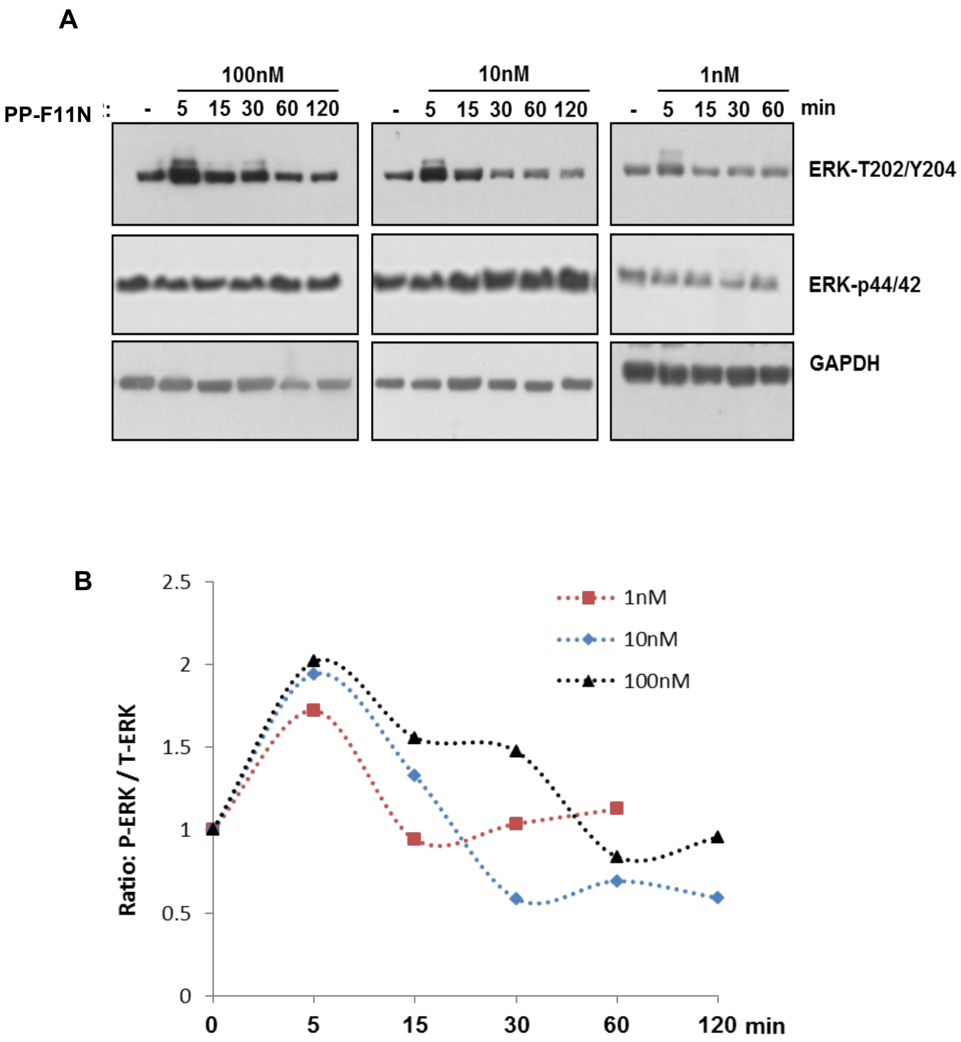


**Figure S3**. **Activation of ERK phosphorylation in response to PP-F11N.** (**A**) Western blot analysis using phosho-ERK1/2-specific antibody on total protein lysates isolated from control and PP-F11N-treated A431/CCKBR cells at different concentration and time points, as indicated. Blots were stripped and re-probed with total-ERK and GAPDH antibody for loading control. (**B**) Corresponding graphs represent relative phosphorylation levels of ERK in response to PP-F11N treatment shown as the ratio of phospho-ERK to total-ERK signals. Ratios in the control untreated cells were normalized to 1.

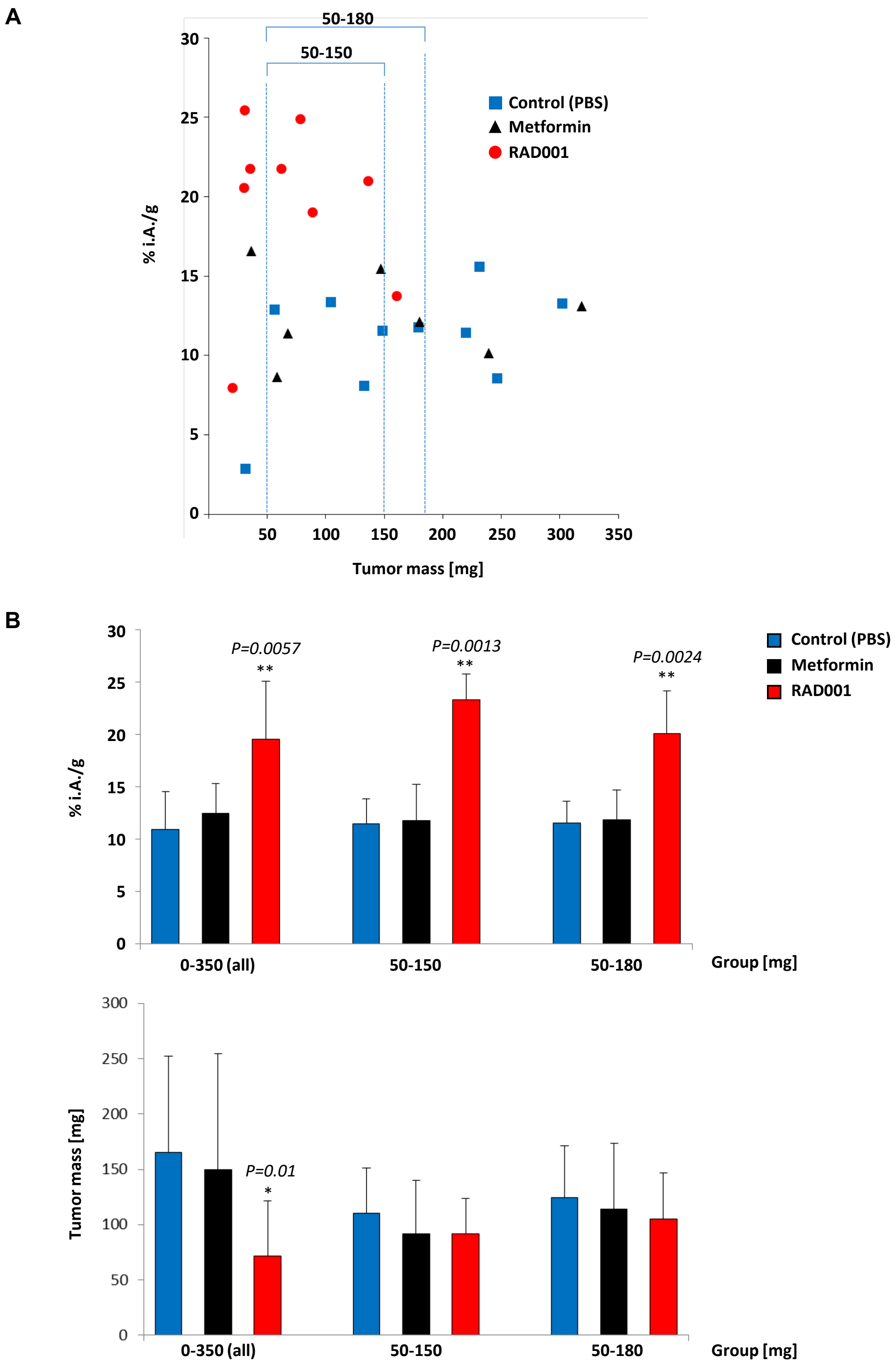


**Figure S4**. **RAD001 increases tumor uptake of radiolabeled minigastrin in tumor-mass-independent manner**. (**A**) Tumor uptake of [$^{177}$Lu]Lu-PP-F11N shown as % of total injected radioactivity per gram of tissue (i.A./g) and tumor masses for all tumors isolated from control, metformin and RAD001-treated mice. The extracted data corresponds to the results shown in Figure 4A. (**B**) Bars represent means of % i.A./g (upper panel) and tumor masses (lower panel) ± SD in three groups including all tumors as well as two groups of tumor masses of 50-150 and 50-180 g.

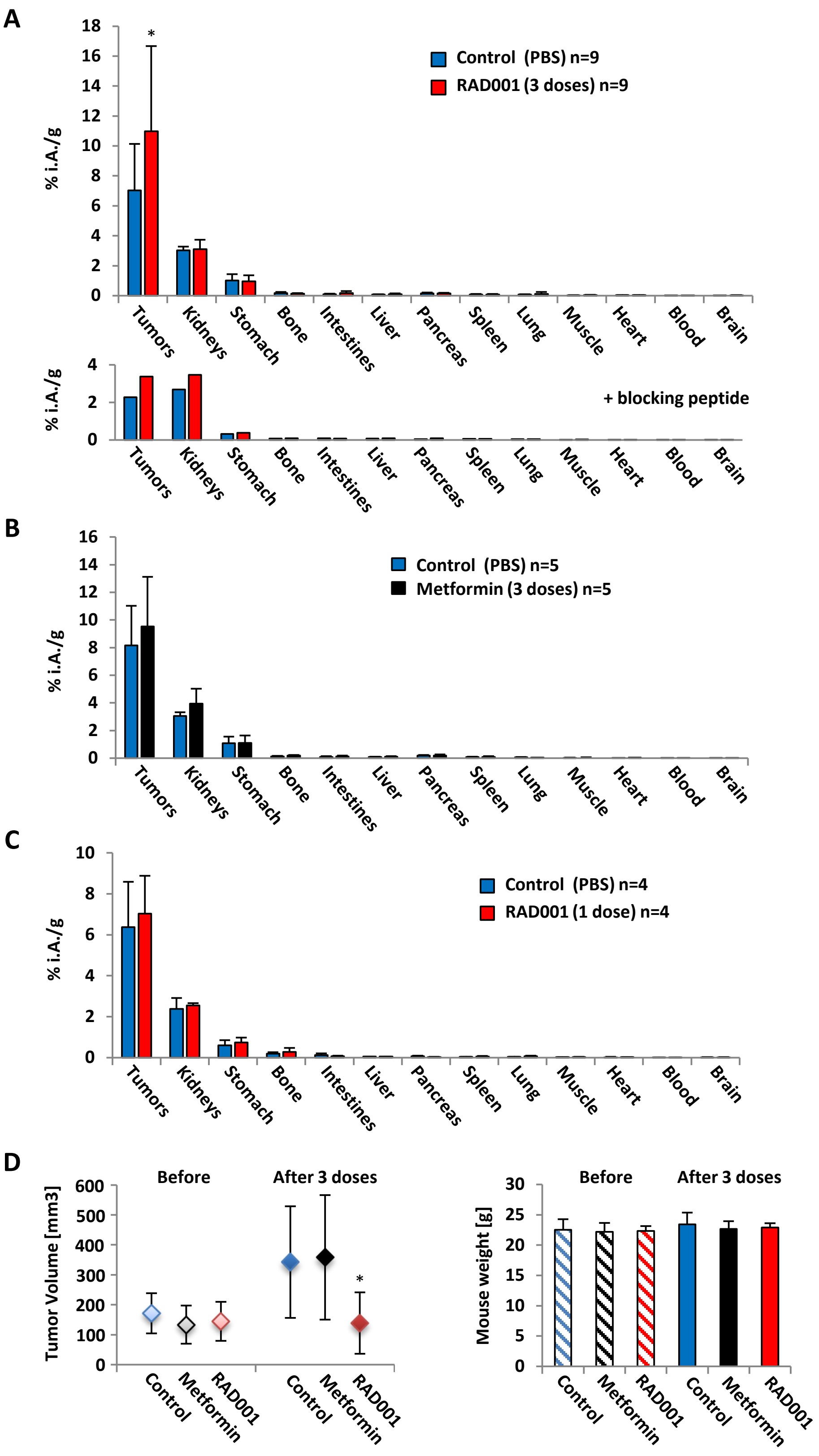


**Figure S5**. **Three daily doses of RAD001 increase CCKBR-dependent tumor uptake of [$^{177}$Lu]Lu-PP-F11N.** A431/CCKBR cells were implanted into immunocompromised mice. RAD001, metformin and control (PBS) animal groups received 3 (**A** and **B**) or 1 (**C**) daily dose and the biodistribution study was accomplished on the next day. Biodistribution of [$^{177}$Lu]Lu-PP-F11N analyzed 4 h after tail vein administration is shown as % of total injected radioactivity per gram of tissue (% i.A./g). Lower panel in A; corresponding biodistribution after co-injection with a blocking peptide. (**D**) Tumor volume and moue weight before and after 3-day treatment. Data represent mean ± SD. **P* < 0.05

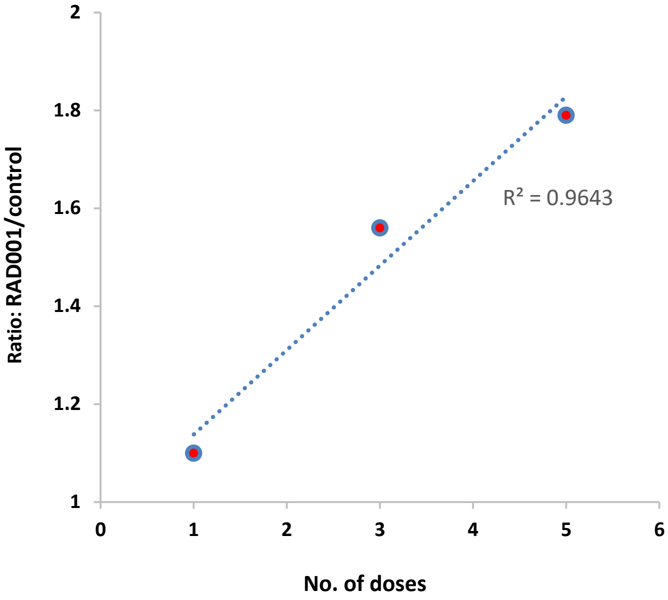


**Figure S6**. **Correlation between number of administrated RAD001 doses and [$^{177}$Lu]Lu-PP-F11N tumor uptake.** A431/CCKBR-tumor bearing nude mice received 1, 3 and 5 daily doses of RAD001 or PBS (control group) and the biodistribution study was accomplished on the next day. The tumor uptakes are shown as the ratios of the means (% of total injected radioactivity per gram of tissue) in RAD001-treated and control PBS-treated mice and correspond to the data shown in Figure 5 and Figure S5A-C.

## Supplementary Tables

| Inhibitor | Radioactivity Uptake [cpm] | SD | *P*-value | Proliferation [$A_{570}$] | SD | *P*-value |
|---|---|---|---|---|---|---|
| Control | 13932 | 856 | - | 0.97 | 0.15 | - |
| BML-257 | 17484 | 701 | 0.0103 | 0.95 | 0.17 | 0.9998 |
| SC-514 | 17433 | 1185 | 0.0123 | 1.01 | 0.17 | 0.9995 |
| Rapamycin | 17297 | 63 | 0.0197 | 0.91 | 0.15 | 0.9993 |
| Daidzein | 16761 | 331 | 0.0993 | 0.92 | 0.10 | 0.9995 |
| AG-494 | 16728 | 2821 | 0.1085 | 0.97 | 0.12 | 0.9999 |
| LFM-A13 | 16712 | 1364 | 0.1131 | 0.93 | 0.08 | 0.9996 |
| Terreic acid | 16496 | 483 | 0.1932 | 1.06 | 0.33 | 0.9986 |
| AG-126 | 16248 | 1308 | 0.3311 | 1.01 | 0.07 | 0.9994 |
| Kenpaullone | 16107 | 240 | 0.4321 | 0.99 | 0.13 | 0.9997 |
| 5,6-dichloro-1-β-D-ribofuranosylbenzimidazole | 16015 | 2987 | 0.5068 | 0.99 | 0.11 | 0.9997 |
| HA-1077·2HCl | 15693 | 602 | 0.7875 | 0.99 | 0.08 | 0.9997 |
| Y-27632·2HCl | 15687 | 1313 | 0.7929 | 1.02 | 0.10 | 0.9993 |
| Genistein | 15630 | 1715 | 0.8371 | 0.95 | 0.09 | 0.9999 |
| HA-1004·HCl | 15602 | 112 | 0.8576 | 1.05 | 0.14 | 0.9988 |
| TYRPHOSTIN 23 | 15599 | 863 | 0.8595 | 1.07 | 0.11 | 0.9985 |
| TYRPHOSTIN 47 | 15570 | 1992 | 0.8791 | 0.98 | 0.07 | 0.9998 |
| Apigenin | 15382 | 589 | 0.9657 | 0.90 | 0.13 | 0.9991 |
| Triciribine | 15335 | 463 | 0.9788 | 0.73 | 0.15 | 0.4253 |
| AG-490 | 15280 | 463 | 0.9813 | 1.03 | 0.1 | 0.9991 |
| SU1498 | 15243 | 904 | 0.9825 | 0.98 | 0.12 | 0.9998 |
| TYRPHOSTIN 25 | 15224 | 817 | 0.9831 | 1.12 | 0.12 | 0.963 |
| Quercetin·2H2O | 15184 | 581 | 0.9845 | 1.03 | 0.10 | 0.9991 |
| H-9·HCl | 15165 | 1524 | 0.9914 | 1.03 | 0.08 | 0.9993 |
| TYRPHOSTIN 46 | 15154 | 178 | 0.9915 | 1.04 | 0.09 | 0.9991 |
| ZM 449829 | 15148 | 2652 | 0.9916 | 0.98 | 0.11 | 0.9999 |
| 2,2',3,3',4,4'-Hexahydroxy-1,1'-biphenyl-6,6'-dimethanol dimethyl ether | 14899 | 1570 | 0.9985 | 0.98 | 0.12 | 0.9998 |
| SB-203580 | 14789 | 1623 | 0.9987 | 0.95 | 0.08 | 0.9998 |
| BAY 11-7082 | 14745 | 1460 | 0.9988 | 1.14 | 0.08 | 0.9157 |
| SB-202190 | 14703 | 1709 | 0.9988 | 1.02 | 0.15 | 0.9993 |
| AG-879 | 14434 | 3197 | 0.9993 | 0.80 | 0.15 | 0.9522 |
| Piceatannol | 14356 | 1981 | 0.9994 | 1.02 | 0.09 | 0.9993 |
| BML-259 | 14290 | 980 | 0.9996 | 1.09 | 0.16 | 0.998 |
| H-7·2HCl | 14068 | 2858 | 0.9998 | 0.98 | 0.07 | 0.9999 |
| AG-825 | 13994 | 1869 | 0.9999 | 0.80 | 0.08 | 0.9444 |
| ZM 336372 | 13988 | 2087 | 0.9999 | 0.87 | 0.11 | 0.9986 |
| Indirubin | 13946 | 2274 | 0.9999 | 1.09 | 0.10 | 0.9919 |
| PD-98059 | 13795 | 238 | 0.9998 | 1.01 | 0.10 | 0.9994 |
| 2-Hydroxy-5-(2,5-dihydroxybenzylamino)benzoic acid | 13793 | 1474 | 0.9998 | 1.01 | 0.13 | 0.9995 |
| Iso-olomoucine | 13789 | 2766 | 0.9998 | 0.96 | 0.08 | 0.9999 |
| AG-370 | 13736 | 1676 | 0.9997 | 0.97 | 0.09 | 0.9999 |
| U-0126 | 13702 | 1064 | 0.9997 | 0.87 | 0.09 | 0.9987 |
| Lavendustin A | 13653 | 1765 | 0.9996 | 1.15 | 0.09 | 0.8692 |
| RG-1462 | 13568 | 2293 | 0.9995 | 0.97 | 0.14 | 0.9999 |
| H-89·2HCl | 13567 | 1559 | 0.9995 | 0.97 | 0.09 | 0.9999 |
| H-8 | 13465 | 951 | 0.9994 | 0.99 | 0.12 | 0.9997 |
| KN-93 | 13425 | 1739 | 0.9993 | 0.92 | 0.12 | 0.9995 |
| TYRPHOSTIN 51 | 13382 | 2157 | 0.9993 | 1.05 | 0.09 | 0.9988 |
| TYRPHOSTIN 1 | 13236 | 1802 | 0.999 | 0.97 | 0.07 | 0.9999 |
| Roscovitine | 12828 | 562 | 0.9981 | 0.91 | 0.09 | 0.9993 |
| TYRPHOSTIN AG 1295 | 12661 | 1322 | 0.9839 | 0.94 | 0.13 | 0.9997 |
| ML-7·HCl | 12659 | 1898 | 0.9838 | 0.95 | 0.09 | 0.9998 |
| TYRPHOSTIN AG 1288 | 12468 | 2123 | 0.9633 | 0.97 | 0.09 | 0.9999 |
| Hydroxy-2-naphthalenylmethylphosphonic acid | 12094 | 1292 | 0.7236 | 1.00 | 0.11 | 0.9996 |
| D-erythro-Sphingosine | 11965 | 2211 | 0.6081 | 0.96 | 0.11 | 0.9999 |
| 2-Aminopurine | 11877 | 700 | 0.5317 | 0.96 | 0.10 | 0.9999 |
| Indirubin-3'-monooxime | 11871 | 1615 | 0.5269 | 0.87 | 0.19 | 0.9986 |
| Olomoucine | 11837 | 1017 | 0.498 | 0.98 | 0.14 | 0.9999 |
| SU 4312 | 11783 | 1252 | 0.4536 | 0.97 | 0.09 | 0.9999 |
| ML-9·HCl | 11729 | 1468 | 0.4116 | 0.94 | 0.09 | 0.9997 |
| GW 5074 | 11704 | 825 | 0.3928 | 0.93 | 0.13 | 0.9996 |
| SP 600125 | 11696 | 2685 | 0.3872 | 0.77 | 0.11 | 0.7709 |

| | | | | | | |
|---|---|---|---|---|---|---|
| AG-1296 | 11524 | 1059 | 0.2745 | 0.94 | 0.11 | 0.9997 |
| GF 109203X | 11524 | 1979 | 0.2742 | 0.95 | 0.09 | 0.9998 |
| N9-isopropyl-olomoucine | 11522 | 920 | 0.2735 | 0.93 | 0.17 | 0.9996 |
| LY 294002 | 11503 | 1594 | 0.2623 | 0.92 | 0.11 | 0.9995 |
| Erbstatin analog | 11451 | 1356 | 0.2342 | 1.12 | 0.17 | 0.9794 |
| KN-62 | 11355 | 1711 | 0.1878 | 0.82 | 0.08 | 0.9816 |
| Palmitoyl-DL-carnitine | 10983 | 1079 | 0.0715 | 0.91 | 0.11 | 0.9993 |
| Wortmannin | 10803 | 1043 | 0.0421 | 0.86 | 0.11 | 0.9984 |
| PP1 | 10656 | 279 | 0.0265 | 0.80 | 0.09 | 0.943 |
| Rottlerin | 10635 | 1653 | 0.0247 | 0.64 | 0.15 | 0.0614 |
| TYRPHOSTIN AG 1478 | 10261 | 2344 | 0.0067 | 0.72 | 0.15 | 0.3955 |
| TYRPHOSTIN 9 | 9839 | 1211 | 0.0013 | 0.62 | 0.10 | 0.0427 |
| Hypericin | 9798 | 742 | 0.0011 | 0.93 | 0.12 | 0.9996 |
| PP2 | 9544 | 1328 | 0.0004 | 0.86 | 0.07 | 0.9984 |
| PKC-412 | 8423 | 685 | 0.0001 | 0.78 | 0.16 | 0.85 |
| Ro 31-8220 mesylate | 8234 | 1456 | 0.0001 | 0.51 | 0.09 | 0.0009 |
| 5-Iodotubericidin | 6929 | 288 | 0.0001 | 1.15 | 0.10 | 0.8273 |
| BML-265 | 6591 | 1044 | 0.0001 | 0.78 | 0.13 | 0.8366 |
| Staurosporine | 1849 | 503 | 0.0001 | 0.51 | 0.09 | 0.001 |

**Table S1**. Mean Uptake of [$^{177}$Lu]Lu-PP-F11N and proliferation rates ±SD in kinase inhibitor-treated and control untreated A431/CCKBR cells.

| Treatment | Organ | Uptake [% i.A./g] | SD |
|---|---|---|---|
| PBS | Tumor | 10.93 | 3.59 |
| Metformin | | 12.48 | 2.81 |
| RAD001 | | 19.56 | 5.53 |
| PBS | Kidney | 3.07 | 0.34 |
| Metformin | | 3.19 | 0.75 |
| RAD001 | | 3.22 | 0.25 |
| PBS | Stomach | 1.01 | 0.38 |
| Met | | 0.97 | 0.40 |
| RAD001 | | 0.80 | 0.27 |
| PBS | Bone | 0.37 | 0.17 |
| Metformin | | 0.25 | 0.06 |
| RAD001 | | 0.19 | 0.11 |
| PBS | Intestines | 0.23 | 0.18 |
| Met | | 0.11 | 0.04 |
| RAD001 | | 0.11 | 0.09 |
| PBS | Liver | 0.17 | 0.003 |
| Metformin | | 0.16 | 0.01 |
| RAD001 | | 0.17 | 0.02 |
| PBS | Pancreas | 0.12 | 0.03 |
| Met | | 0.09 | 0.01 |
| RAD001 | | 0.17 | 0.07 |
| PBS | Spleen | 0.10 | 0.02 |
| Metformin | | 0.08 | 0.004 |
| RAD001 | | 0.08 | 0.02 |
| PBS | Lung | 0.08 | 0.01 |
| Met | | 0.05 | 0.007 |
| RAD001 | | 0.06 | 0.005 |
| PBS | Muscle | 0.06 | 0.06 |
| Metformin | | 0.03 | 0.01 |
| RAD001 | | 0.05 | 0.03 |
| PBS | Heart | 0.05 | 0.02 |
| Metformin | | 0.04 | 0.01 |
| RAD001 | | 0.031 | 0.006 |
| PBS | Blood | 0.043 | 0.008 |
| Metformin | | 0.03 | 0.01 |
| RAD001 | | 0.04 | 0.008 |
| PBS | Brain | 0.03 | 0.04 |
| Metformin | | 0.007 | 0.002 |
| RAD001 | | 0.008 | 0.003 |

**Table S2**. Biodistribution of [$^{177}$Lu]Lu-PP-F11N ± SD shown as % of total injected radioactivity per gram of tissue (% i.A./g) 4 h post injection in RAD001, Metformin and control PBS-treated A431/CCKBR-tumor bearing nude mice.
